# Prediction of global potential distribution and assessment of habitat suitability for *Xanthium spinosum* driven by climate change

**DOI:** 10.3389/fpls.2025.1690546

**Published:** 2025-11-17

**Authors:** Yuke Fan, Xiaowei Zhang, Junlong Yang, Jun Yang, Hongmei Zhang, Bo Yang, Xiaowei Li

**Affiliations:** 1 College of Forestry and Prataculture, Ningxia University, Yinchuan, China; 2 College of Forestry, Gansu Agricultural University, Lanzhou, China

**Keywords:** alien invasive plants, *Xanthium spinosum*, climate change, potential distribution, species distribution models

## Abstract

*Xanthium spinosum* Linn (Asteraceae family), native to South America, is among the most invasive plant species globally, with major ecological, agricultural, and livestock-related impacts. However, little is known about how climate change may alter its future distribution and range shifts. This study assessed the potential global distribution and habitat suitability of *X. spinosum* by evaluating its dispersal risk under climate change. We compiled 13,378 global occurrence records and applied the MaxEnt model (optimized via the R package ENMeval) to simulate habitat suitability under current conditions and three future climate scenarios (SSP126, SSP370, and SSP585) for 2040–2060, 2060–2080, and 2080–2100. The model performed with high accuracy (area under the curve > 0.979). The most influential factor was the minimum temperature of the coldest month (Bio6; 67.1% contribution), with an optimal range of −7.3 °C to 8.7 °C. Other key drivers included Bio10, Bio19, and Bio7. Currently, core suitable areas include western North America to central/western Europe, southeastern South America to West Africa, and Southeastern Australia to East Asia, spanning 2,950.42 × 10^4^ km^2^ (52.8% of potential distribution). Under SSP126, suitable habitats expand steadily (+338.15 × 10^4^ km^2^ by 2080–2100). SSP370 projects large fluctuations, peaking at + 448.26 × 10^4^ km^2^ in 2060–2080. SSP585 predicts rapid early expansion (+392.54 × 10^4^ km^2^ by 2040–2060), with the rate of expansion decreasing in the mid and late stages. These findings support invasion risk assessment, early warning development, and targeted management strategies for *X. spinosum* in a changing climate.

## 1 Introduction

Invasive alien species are a critical component of global environmental change that pose substantial threats to national biosecurity, ecological security, and food security (Seebens et al., 2017; Van Kleunen et al., 2015; Guo et al., 2025). These invaders typically exhibit rapid growth, high resource use efficiency, strong dispersal capacity, and enhanced resistance to pathogens (Máximo et al., 2020). Through interference mechanisms, such as hybridization, alteration of natural habitats, and introduction of pathogenic organisms, they achieve a competitive advantage over native species, driving the latter toward decline or extinction, thereby occupying their ecological niches to facilitate self-expansion (Baldi et al., 2022). Under intensifying global climate change, phenomena including warming climate, elevated atmospheric CO_2_ concentrations, increased nitrogen deposition, and frequent extreme climatic events have profoundly affected global ecosystem stability. Such changes drive species adaptation shifts, further exacerbating the dispersal risks of invasive alien species (Blackburn et al., 2011; Ren et al., 2022). Invasive plants are a primary cause of biodiversity loss. Compared with native flora, they exhibit significant competitive advantages in light acquisition, soil moisture utilization, and nutrient exploitation (Adkins and Shabbir, 2014; Karimmojeni et al., 2021). Once successfully established, highly adaptable and dispersive alien species trigger drastic declines in native biodiversity. Their effects extend beyond the direct displacement of indigenous species to fundamentally alter ecosystem structure and function, destroying habitats for multiple species and thereby posing severe threats to biodiversity (Zheng and Li, 2025).

Although native to subtropical South America, *X. spinosum* has become naturalized as a wild species across Central Europe, Southern Europe, the northwestern Pacific (Martin and Carnahan, 1983), Asia, and North America (Löve and Dansereau, 1959; Gligor et al., 2022). It is a toxic invasive annual weed (Wang et al., 2025) that is listed by the European Plant Protection Organization (EPPO) as a highly invasive plant species (EPPO Global Database, 2025). Its traits include extended flowering periods, high fecundity, prolific seed production, and strong cold tolerance (Yuan et al., 2018; Kelečević et al., 2024). Its seeds have hooked spines, which readily attach to livestock (e.g., cattle and sheep) and cargo, facilitating dispersal. This not only reduces the economic value of animal wool and hides but also drives large-scale spread of *X. spinosum* through adaptability to Mediterranean climates and affinity for nitrogen-rich soils (Andreani et al., 2017). *X. spinosum* has achieved global distribution (Weber, 2017), and the corresponding effects are particularly severe in ecologically fragile regions with simplified ecosystem structures (Wang et al., 2025). It suppresses native plants via allelopathy and, upon successful establishment, damages various crops (Liu et al., 2025). Consequently, it severely threatens biodiversity, agricultural production, and livestock farming in invaded areas while substantially increasing ecological restoration costs and economic management burden. Therefore, effective control of *X.* *spinosum* represents a major challenge.

The capacity of species to respond to climate change partly depends on their ability to disperse into suitable habitats, which are subject to climate change effects (Travis et al., 2013). Current research on invasive species indicate that climate change will considerably alter habitat suitability for invasive species, particularly considering the projected continuous increase in global mean temperatures through 2100 (Aidoo et al., 2025). The potential distribution of *X. spinosum* may shift, which is a critical question needing consideration. Consequently, predicting temporal changes in the potential distributions of invasive species is essential for effective management. Species distribution models (SDMs) can simulate future distribution scenarios, thereby support invasion risk reduction and optimizing conservation efforts (Guisan and Zimmermann, 2000); however, limited research on habitat suitability for *X. spinosum* hinders the formulation of early monitoring and prevention strategies. Previous studies have investigated *X. spinosum* primarily in terms of bio-ecological traits (Liu et al., 2025), invasion mechanisms and dispersal patterns (Xiao et al., 2023), isolation and identification of bioactive compounds, and allelopathic effects of secondary metabolites (Yuan et al., 2018). However, SDM-based predictions of suitable habitats for *X. spinosum* during climate change are scarce, with no existing global analysis. Therefore, projecting the climate-driven global geographic distribution patterns of *X.* *spinosum* is critically important.

SDMs serve as vital tools for studying how suitable habitats of species respond to climatic and environmental changes (Wiens et al., 2009; Hosseini et al., 2024). These models use geographic distribution data obtained from field surveys, herbarium records, and literature sources to infer the ecological niches of species. Based on the density patterns of occurrence points, they quantify the habitat preferences of species through probability estimates, thereby predicting the response of potentially suitable niches to climate change (Bellard et al., 2012). Commonly used SDM methods include bioclimatic envelope models (BIOCLIM) (Booth et al., 2014), maximum entropy (MaxEnt) models (Phillips et al., 2006), genetic algorithm for rule-set production models (Stockwell, 1999), generalized additive models (GAMs) (Hastie and Tibshirani, 1987), and generalized linear models (GLMs) (Nelder and Wedderburn, 1972; Beaumont et al., 2008). Among these models, the MaxEnt model demonstrates superior tolerance to sample bias and higher predictive accuracy compared with other models, yielding relatively optimal performance (Elith et al., 2006; Merow et al., 2014). Consequently, it has been widely used for predicting species distributions (Merow et al., 2013).

In this study, we elucidated the distribution patterns and dynamic changes of potentially suitable habitats for *X. spinosum*, providing scientific references for global invasion risk assessment, early warning systems, and management strategies. Under global climate change, we integrated global occurrence records of *X.* *spinosum* with multidimensional environmental factors. Using an optimized MaxEnt model (implemented via the R package ENMeval) combined with ArcGIS software, we simulated its potential suitable habitats under current conditions and three future periods (2040–2060, 2060–2080, and 2080–2100) across three Shared Socioeconomic Pathways (SSP126, SSP370, and SSP585). Through regional-scale suitability classification and an analysis of key environmental drivers, the results identify current high-risk invasion hotspots, predict future habitat expansion trends, and inform targeted control measures, thereby offering critical scientific guidance for global management.

## 2 Materials and methods

### 2.1 Collection of species distribution data

Global distribution records of *X. spinosum* were compiled from multiple sources: the iNaturalist (iNat, https://www.inaturalist.org/), the Global Biodiversity Information Facility (GBIF, https://www.gbif.org/), relevant literature. For specimen records lacking latitude and longitude coordinates, precise geographic coordinates were obtained using the Baidu Map Coordinate Picker System (http://api.map.baidu.com/lbsapi/getpoint/index.html). A preliminary total of 25,245 distribution records were collected (iNat: 4528; GBIF: 20,685; literature: 32). To ensure high-quality data, a rigorous screening of the raw records was performed. Records with errors, those falling outside of the species’ actual distribution range, or those lacking specific geographic information were excluded. Because data from different sources (databases, literature) may exhibit spatial clustering (i.e., multiple records at adjacent locations), which can easily cause spatial autocorrelation in SDMs, leading to model overfitting and reduced predictive accuracy (Teng et al., 2018), we applied spatial thinning to the data using the ENMTools package in R (Warren et al., 2021). In particular, only one unique distribution point was retained within a 20-km radius. This mitigated spatial autocorrelation and considerably improved the accuracy of the model predictions for the potential suitable geographic distribution of *X. spinosum*. Ultimately, 9,134 valid distribution points with latitude and longitude data were obtained. These data were organized, imported into Excel, and saved in CSV format for subsequent model construction.

### 2.2 Collection of environmental variables and setting of future climate scenarios

This study incorporated two categories of environmental variables: climate and soil are shown in **Table 1**. Nineteen bioclimatic variables (Bio1–Bio19) were sourced from the WorldClim 2.1 database (https://worldclim.org/) at a spatial resolution of 2.5 arc-minutes. Ten soil factors were obtained from the Harmonized World Soil Database (HWSD) v2.0 (https://www.fao.org/). After model construction, the projections of the potential suitable habitats of *X. spinosum* under future climate scenarios (2040–2060, 2060–2080, and 2080–2100) used future-period climate data while maintaining current soil conditions. All environmental layers were standardized to 2.5 arc-minute resolution. Because CMIP6 (Coupled Model Intercomparison Project Phase 6) integrates Shared Socioeconomic Pathways (SSPs) with socioeconomic factors, considerably enhancing the scientific robustness of future simulations (Popp et al., 2017; Khadka et al., 2022), we adopted future bioclimatic data from the EC-Earth3 climate model under CMIP6, which is well-regarded for simulations over European regions. Three representative SSP scenarios were selected to reflect progressively elevated carbon emissions: SSP126 (Sustainable Development Pathway), SSP370 (Intermediate Development Pathway), and SSP585 (Rapid Development Pathway).

**Table 1:** Description of environmental variables.

Type	Field	Description and unit	Type	Field	Description and unit
Climatic factors	Bio1	Annual mean temperature °C		Bio16	Precipitation of the wettest quarter (mm)
	Bio2^*^	Mean diurnal range °C		Bio17^*^	Precipitation of the driest quarter (mm)
	Bio3^*^	Isothermality		Bio18^*^	Precipitation of the warmest quarter (mm)
	Bio4	Temperature seasonality		Bio19^*^	Precipitation of the coldest quarter (mm)
	Bio5	Max temperature of the warmest month °C	Soil factors	t-Ece	Soil conductivity dS/m
	Bio6^*^	Min temperature of the coldest month °C		t-Clay	Clay content %wt
	Bio7^*^	Temperature annual range °C		t-Cec-Clay	Soil cation exchange content cmol/kg
	Bio8^*^	Mean temperature of the wettest quarter °C		t-CaSO_4_^*^	Soil sulfate content %weight
	Bio9^*^	Mean temperature of the driest quarter °C		t-CaCO_3_^*^	Soil carbonate content %weight
	Bio10^*^	Mean temperature of the warmest quarter °C		t-Gravel	Soil gravel content %vol.
	Bio11	Mean temperature of the coldest quarter °C		t-Sand	Sand content %wt.
	Bio12^*^	Annual precipitation (mm)		t-Silt	Silt content %wt.
	Bio13	Precipitation of the wettest month (mm)		t-Teb	Soil exchangeable salts cmol/kg
	Bio14	Precipitation of the driest month (mm)		t-Esp	Exchangeable sodium salt %
	Bio15^*^	Precipitation seasonality		t-Texture	Topsoil texture

Environmental factors marked with * are variables used in MaxEnt model predictions. Attributes prefixed with “t-” represent upper soil attributes (0–30 cm).

### 2.3 Environmental variable screening

**Figure 1:**
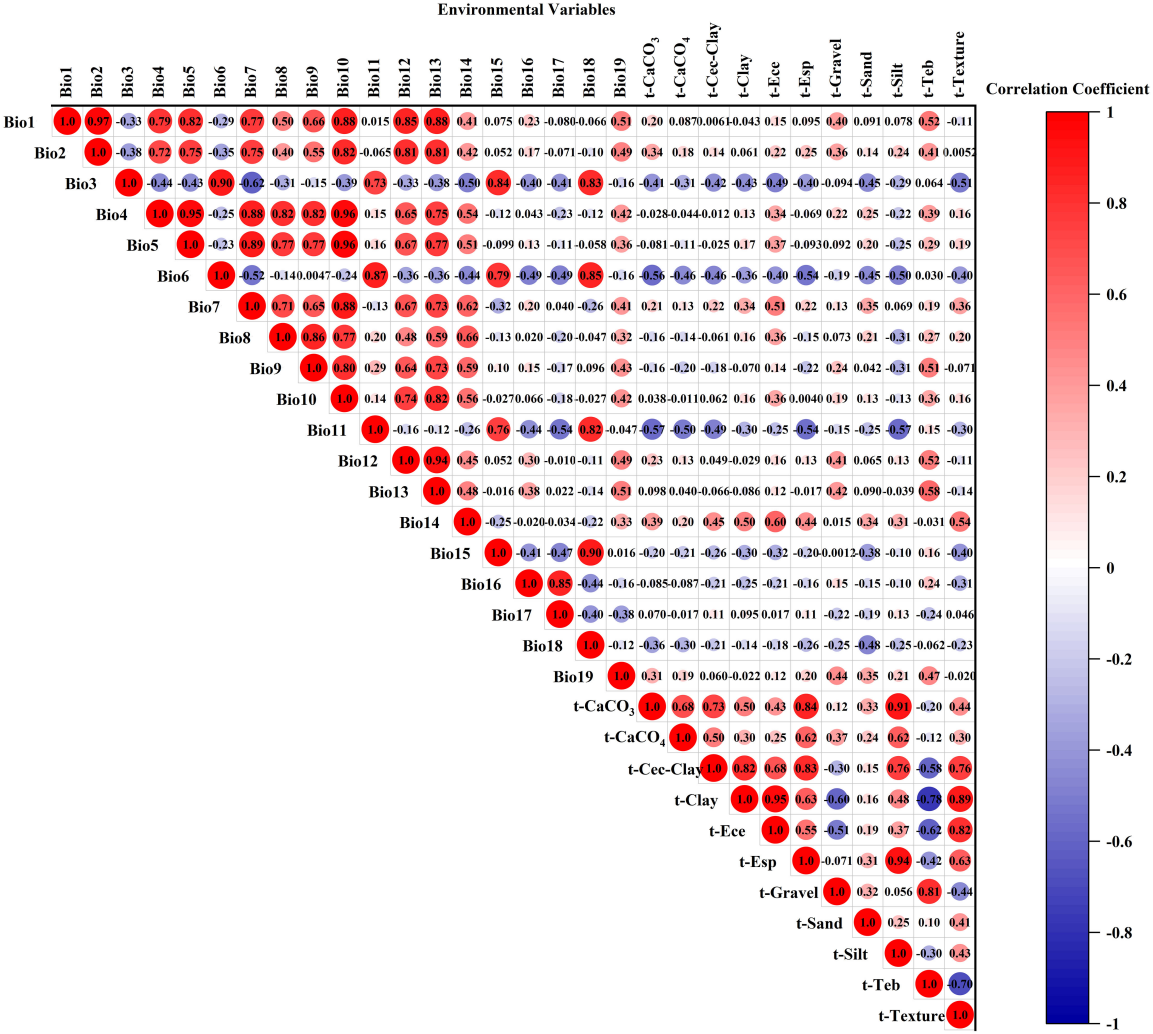
Heatmap of environmental variable correlations.

High correlations among environmental variables can result in overfitting in MaxEnt model results, which compromises the accuracy of variable contribution rates and predicted species distribution ranges (Evans et al., 2010). We implemented the following screening protocol: Preliminary Contribution Screening: All environmental variables were imported into MaxEnt 3.4.4. After 10 replicated runs (cross-validation), the variables were ranked based on their average contribution rates. Variables with a zero-contribution rate were excluded. Multicollinearity Check: Pearson correlation coefficients among retained variables were calculated using the raster package in R, as shown in **Figure 1**. Variables were retained only if absolute correlation coefficients were below 0.8 (|r| < 0.8). For variable pairs with |r| ≥ 0.8, only the variable exhibiting a higher contribution rate was retained (Sharma et al., 2018). Through this process, 14 environmental variables were ultimately selected for modeling.

### 2.4 MaxEnt model parameter optimization

**Figure 2:**
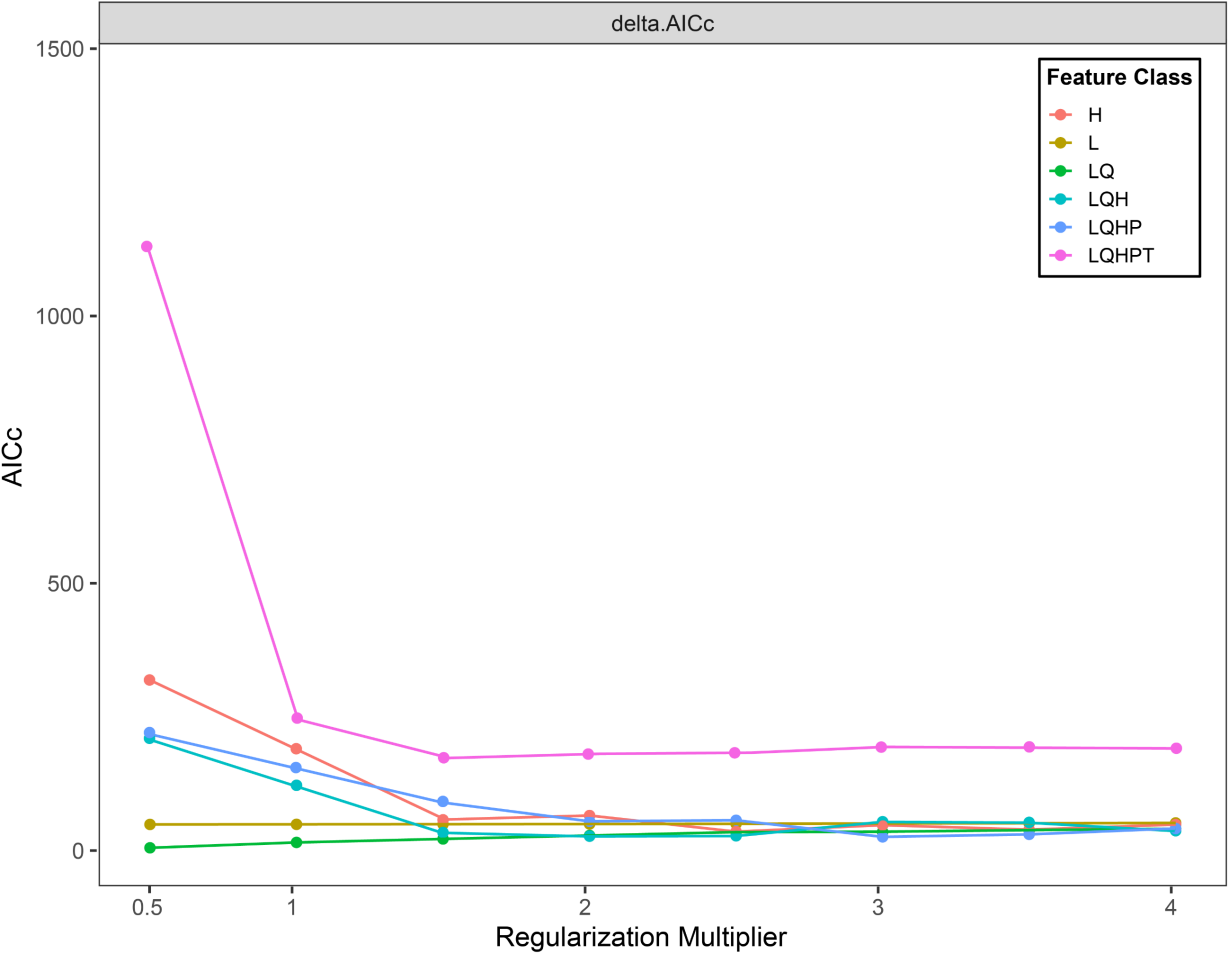
ENMeval packet optimization results.

Using default parameters for habitat prediction across species increases model sensitivity to sampling bias and risks overfitting, thus compromising prediction reliability. Regularization Multiplier (RM) and Feature Class (FC) influence model complexity and predictive accuracy. RM determines model complexity, while FC governs potential shapes of marginal response curves (Phillips et al., 2017). To enhance prediction precision, we used the ENMeval package in R to optimize RM and FC parameters. Model complexity was evaluated using corrected Akaike Information Criterion (AICc) values under different parameter combinations, with those yielding the lowest complexity selected for modeling (Muscarella et al., 2014). Default settings included RM = 1. Standard FC selection proceeded as follows: Linear (L) features always included, Quadratic (Q) features enabled at ≥ 10 occurrence points, Hinge (H) features enabled at ≥ 15 points, Threshold (T) and Product (P) features enabled at ≥ 80 points (West et al., 2016). We tested RM values from 0.5 to 4 (increments of 0.5) and six FC combinations: H, L, LQ, LQH, LQHP, and LQHPT. Based on R analysis, optimized parameters were set to RM = 0.5 and FC=LQ for final model execution as shown in **Figure 2**.

### 2.5 Model training and accuracy evaluation

Following data preparation and model optimization, 13,378 screened distribution points and 14 environmental variables were entered into the optimized MaxEnt model (Version 3.4.4). The dataset was partitioned with 75% of samples for training and 25% for testing (Zhang et al., 2018). Climate variable importance was quantified via jackknife tests and contribution rates (Wan et al., 2019). Model Parameters: maximum background points: 10,000 maximum iterations: 5,000 10-replicate runs to reduce stochastic error (Santana et al., 2019), model performance was evaluated using the AUC of the receiver operating characteristic (ROC) (Liu et al., 2021). AUC values range from 0 to 1, with predictive accuracy classified as Unacceptable (0.50—0.70), Moderate (0.70—0.80), Good (0.80—0.90), Excellent (0.90—1.00) (Araújo et al., 2005), identical parameter configurations were applied for future scenario projections.

### 2.6 Delineation of potential suitable habitats and distribution dynamics

Habitat suitability probabilities derived from the optimized MaxEnt model were classified into suitability levels using the Natural Breaks method (minimizing within-group variance, while maximizing between-group variance) (Ke et al., 2023) in ArcGIS 10.8. Based on the observed distribution of *X. spinosum*, four suitability classes were defined: Unsuitable (0.00–0.10), Low suitability (0.10–0.33), Moderate suitability (0.33–0.64), and High suitability (0.64–1.00). Identical thresholds were applied to future scenarios. Areas with an occurrence probability ≥ 0.33 demonstrated a higher likelihood of species survival (Zhang et al., 2025). Consequently, moderate and high suitability zones were combined as core suitable habitats. The Reclassify tool in ArcGIS quantified areal extents of each suitability class per scenario. Regions with an occurrence probability >0.10 were mapped as potential distribution areas. Species probability maps (0–1 scale) were converted to binary presence/absence maps (0/1) using the ArcGIS con function, with thresholds determined by sensitivity–specificity optimization (Li et al., 2020). Within ArcGIS, the SDMtoolbox 2.5 extension was used to classify habitat dynamics into expansion, stability, and contraction, calculate centroid coordinates for future periods, and trace centroid migration trajectories relative to current conditions.

## 3 Results and analysis

### 3.1 Model predictions and performance evaluation

**Figure 3:**
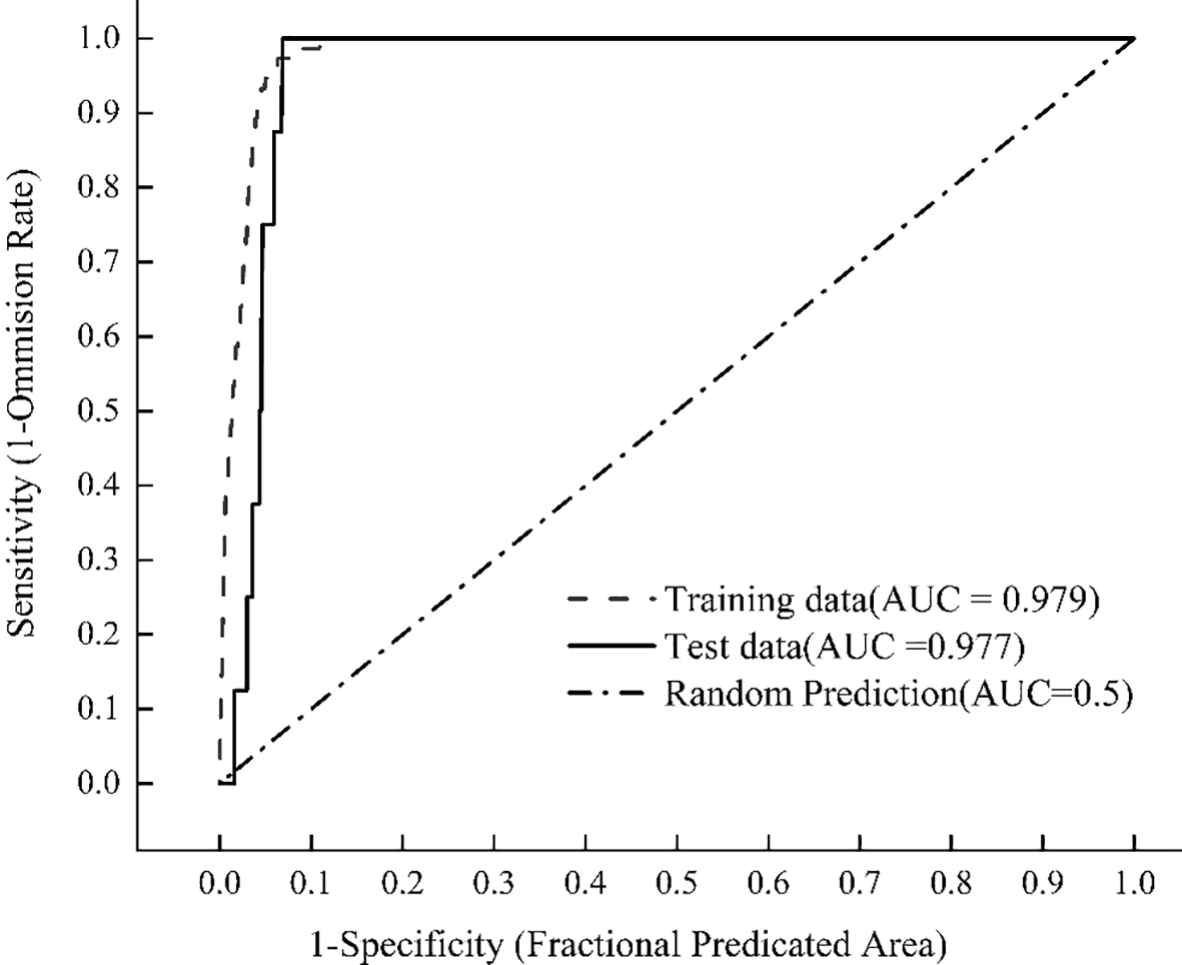
AUC values under the ROC curve.

Model accuracy depends on sample coverage, with AUC representing the optimal evaluation metric (Hosseini et al., 2024). The optimized MaxEnt model simulated the distribution of *X. spinosum* showed that the AUC value of the model training set reached 0.979, as shown in **Figure 3**. These results indicate excellent model fit and a robust predictive capacity for the potential habitats of *X. spinosum*, which confirms the model’s reliability for projecting future distributions.

### 3.2 Dominant factors in habitat suitability

**Figure 4:**
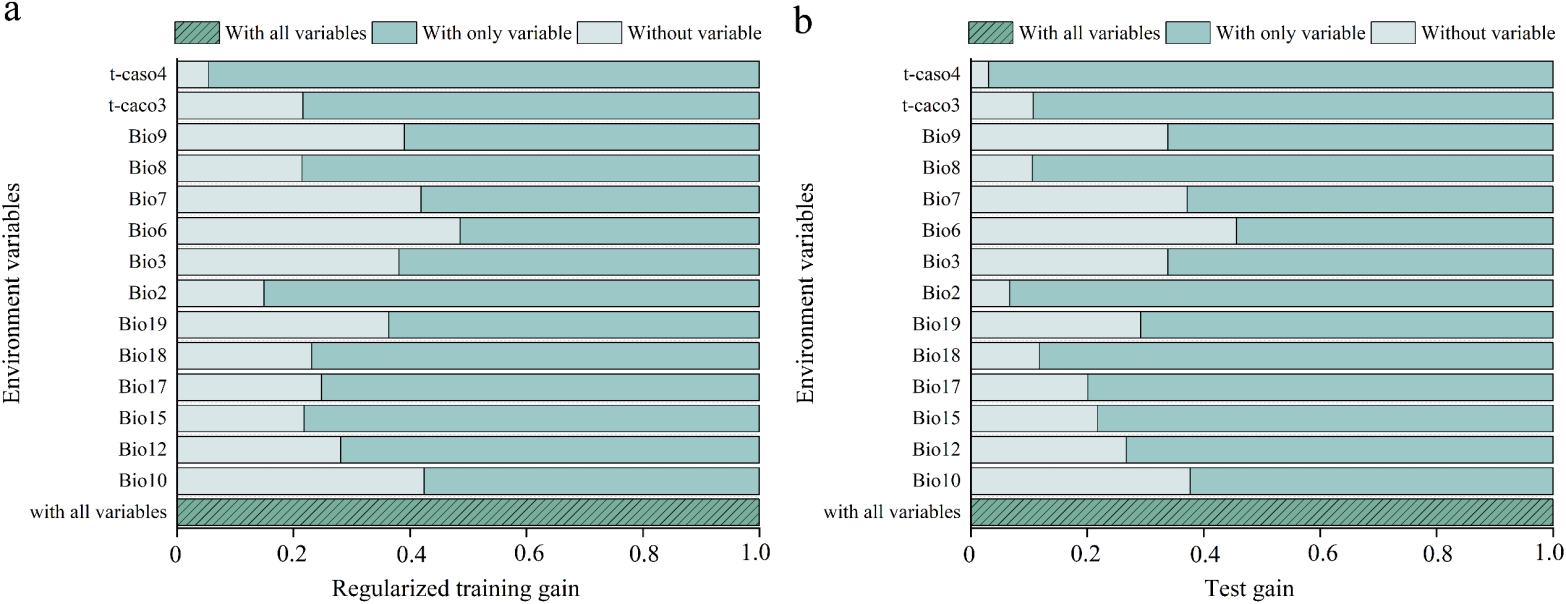
Jackknife test of the MaxEnt model showing the **(a)** regularized training and **(b)** test gains.

This study analyzed the contributions of environmental variables to model construction are shown in **Table 2**. Based on the percent contribution, the top three key variables influencing *X. spinosum* distribution were Minimum Temperature of Coldest Month (Bio6, 67.1%), Precipitation of Coldest Quarter (Bio19, 21.8%), and Mean Temperature of Warmest Quarter (Bio10, 4.1%). These three variables collectively accounted for 93.0% of the total contribution, with Bio6 alone contributing 67.1%, highlighting the dominant role of bioclimatic variables. Jackknife tests further validated variable importance, as shown in **Figure 4**. The results indicated that for light green bars (single variable), higher training gain indicates greater information content and stronger influence on species distribution. For dark green bars (excluding single variables), lower training gain signifies greater uniqueness and importance of the removed variable. Based on the jackknife evaluation of regularized training gain and test gain, the three most critical variables affecting *X. spinosum* distribution were Minimum Temperature of Coldest Month (Bio6), Mean Temperature of Warmest Quarter (Bio10), and Temperature Annual Range (Bio7). Synthesis of percent contribution and jackknife results demonstrated that bioclimatic variables (Bio6, Bio10, Bio19, Bio7) overwhelmingly dominated soil factors in MaxEnt predictions of *X. spinosum* distribution. Bio6 (Minimum Temperature of Coldest Month), Bio10 (Mean Temperature of Warmest Quarter), Bio19 (Precipitation of Coldest Quarter), and Bio7 (Temperature Annual Range) were identified as the key environmental variables for predictive modeling, with Bio6 being the dominant factor because of its significantly higher contribution rate.

**Table 2:** Percent contribution of key environmental variables.

Code	Current/%	2040—2060/%			2060—2080/%			2080—2100/%		
		SSP126	SSP370	SSP585	SSP126	SSP370	SSP585	SSP126	SSP370	SSP585
Bio6	67.1	68.9	61.2	65.1	69.8	57.4	58.9	66.2	61.2	65.8
Bio19	21.8	20.1	20.9	19.5	17.0	20.9	23.7	17.3	22.9	16.5
Bio10	4.1	4.2	5.1	4.8	5.3	3.3	5.3	4.2	6.1	4.2
Bio7	1.3	0.9	1.2	1.0	2.2	1.2	2.3	2.5	2.2	1.7

**Figure 5:**
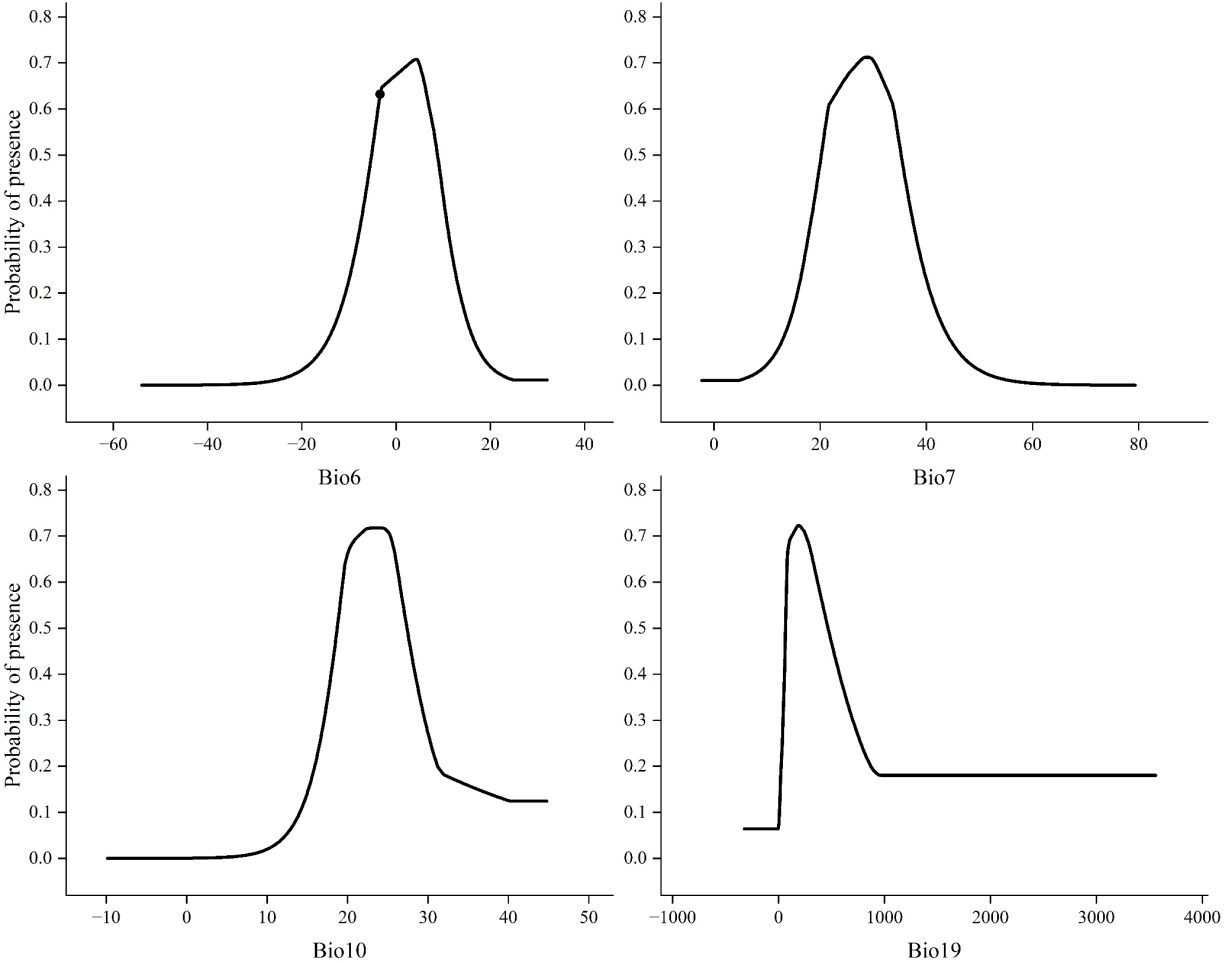
Response curves of key environmental factors.

By analyzing the response curves of environmental factors, the relationship between the survival probability of the target species and specific environmental factors can be determined. A higher survival probability (P) is more conducive to the survival of the species (Zhang et al., 2025). This study defines areas with a survival probability of >0.64 as highly suitable habitats. Therefore, this threshold indicates that the corresponding combination of environmental factors is more favorable for the growth of the species. Analysis of the response curves for the four key environmental factors (Bio6, Bio7, Bio10, Bio19), as shown in **Figure 5** showed the Minimum Temperature of the Coldest Month (Bio6): The suitable range for *X. spinosum* survival (P > 0.64) was −7.3°C to 8.7°C. Survival probability drops below 0.64 when temperatures fall below −7.3°C or rise above 8.7°C. Its optimal growth temperature is approximately 4.2°C (where P peaks). Temperature Annual Range (Bio7): The suitable survival threshold was −22.4°C to 33.2°C. The optimal value was approximately 27.5°C (where P peaks). Mean Temperature of Warmest Quarter (Bio10): The suitable range was narrower (18.3°C to 24.6°C). The optimal temperature was approximately 24.8°C (where P peaks). Precipitation of the Coldest Quarter (Bio19): The suitable range was wider (83.45 mm to 421.83 mm). The optimal precipitation was approximately 207.8 mm (where P peaks).

### 3.3 Suitable habitat distribution of *Xanthium spinosum* under the current climatic scenario

**Figure 6:**
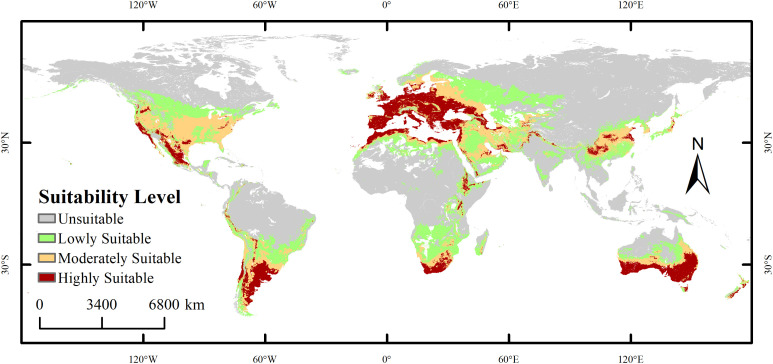
Global distribution of *Xanthium spinosum* under the current climatic scenario.

Under current climatic conditions, the suitable habitat of the species exhibits a significant spatial differentiation pattern, as shown in **Figure 6**. The potential distribution area of *X. spinosum* (occurrence probability >0.1) is widely distributed across central to western North America (120°W–60°W), northern to southeastern South America (60°W–0°), central–western Europe (0°–60°E), central to West Africa (0°–40°E), inland to coastal Asia (60°E–120°E), and southeastern Australia (120°E–180°E), to form a continuous distribution belt spanning five continents, with a total area of 5,620.18 × 10^4^ km². The highly suitable habitat (area 1,380.65×10^4^ km²) exhibits a fragmented, patchy, and banded distribution pattern, with core concentrations near 30°N in 120°W–60°W (western North America) and 0°–60°E (central–western Europe), and near 40°S in 120°E–180°E (southeastern coastal Australia). The moderately suitable habitat (1,569.77×10^4^ km²), serving as a transition zone surrounding the highly suitable areas, is mainly distributed in 60°W–0° (southeastern South America, West Africa) and 60°E–120°E (coastal East Asia and eastern India), showing locally contiguous features. The lowly suitable habitat (2,669.76×10^4^ km²) extensively and continuously covers central North America (60°W–120°W), northern South America (60°W–0°), central Africa (0°–40°E), and inland Asia (60°E–120°E). The unsuitable habitat (8,473.62×10^4^ km²) is widely distributed in high-latitude polar regions (>40°N/S), arid inland areas (e.g., western North America, Central Asia), and core tropical rainforest zones (0°–10° latitude).

### 3.4 Changes in global distribution under three future climate scenarios

**Figure 7:**
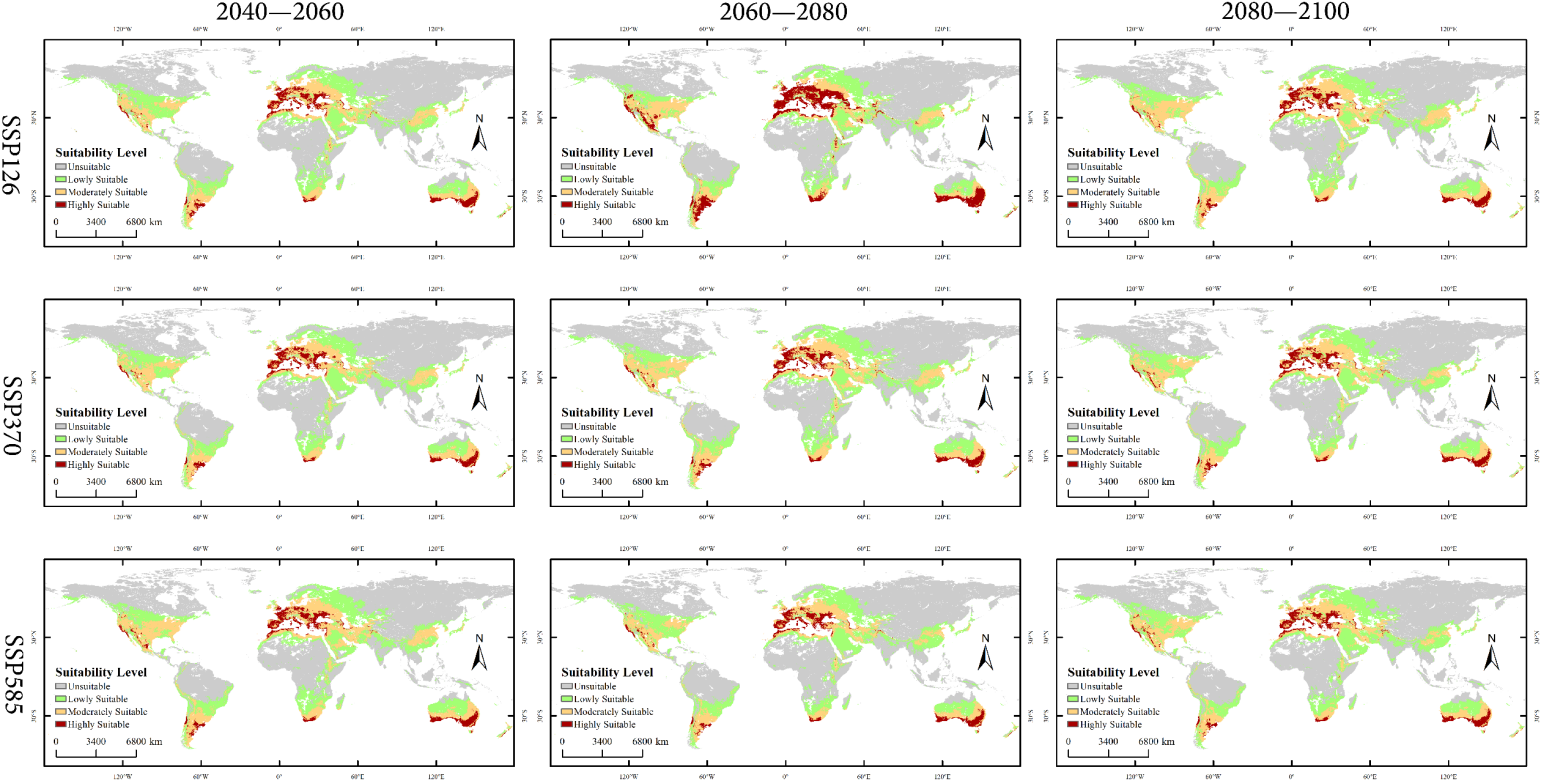
Global distribution of *Xanthium spinosum* under current and future climatic scenarios.

Under the three future climate scenarios (SSP126, SSP370, SSP585), the spatial pattern of suitable habitats for *X. spinosum* undergoes significant changes, with a pronounced displacement of the distribution centroid, as shown in **Figure 7**. Overall, the total suitable habitat area expands compared with the current climate scenario, whereas the core suitable habitat (highly suitable area) generally contracts.

**Figure 8:**
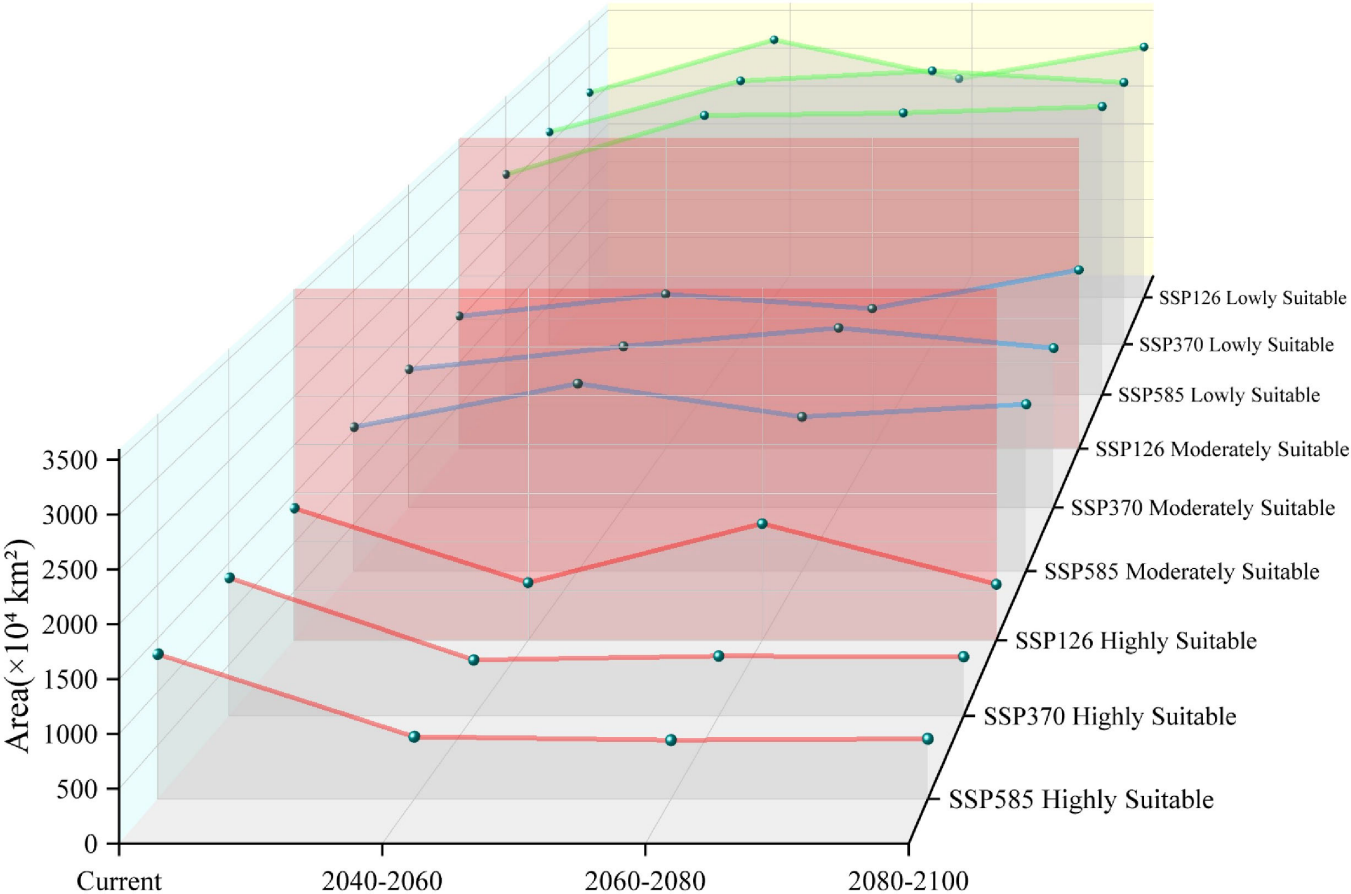
Area by suitability level for *Xanthium spinosum* under current and future climate scenarios.

**Figure 9:**
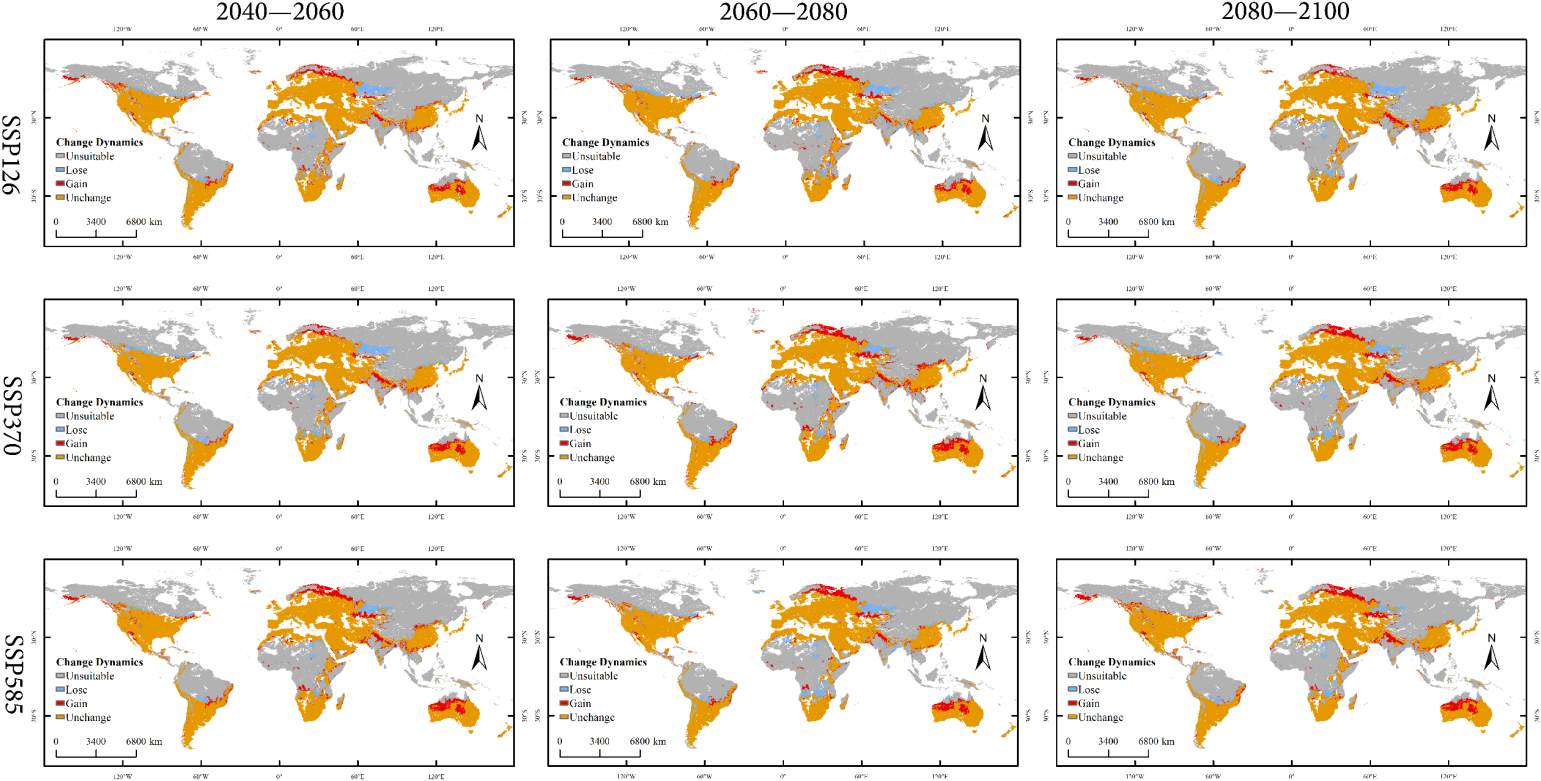
Changes in global spatial distribution patterns of *Xanthium spinosum* under current and future climate scenarios.

Low-emission pathway (SSP126): The suitable habitat area continuously expands over time, with the net increase rising from 170.18 × 10^4^ km² in 2040–2060 to 338.15 × 10^4^ km² in 2080–2100, as shown in **Figure 8**. The core suitable habitat area exhibits the most significant reduction initially (−495.32 × 10^4^ km²). The stable area decreases slightly in the mid-term (−4.77%), recovering to 5105.61 × 10^4^ km² by the end-term, as shown in **Figure 9**, **Table 3**. Medium-emission pathway (SSP370): The suitable habitat area change shows strong fluctuations. The net change shifts from an early increase (+89.74 × 10^4^ km²) to a peak expansion during the mid-term (+448.26 × 10^4^ km²), then returns to an increase by the end-term (+82.56 × 10^4^ km²). The core suitable habitat area exhibits the most pronounced reduction initially (−535.21 × 10^4^ km²). The stable area begins to expand from the mid-term (+151.17 × 10^4^ km²), but decreases slightly by the end-term (−0.70%), with the 2080–2100 stable area being 4905.94 × 10^4^ km². Under the high-emission pathway (SSP585), suitable habitat area has an early peak in net increase (+392.54 × 10^4^ km²), exhibits a sharp slowdown in expansion mid-term (+46.85 × 10^4^ km²), and returns to an increase by the end-term (+265.38 × 10^4^ km²). The stable area continuously decreases; the largest reduction occurs mid-term (−4.00%) and the area shrinks to 4995.85 × 10^4^ km² by the end-term.

**Table 3:** Changes in global suitable habitat area of *Xanthium spinosum* under current and future climate scenarios.

Change Dynamics	2040—2060 × 10^4^ km²			2060—2080 × 10^4^ km²			2080—2100 × 10^4^ km²		
	SSP126	SSP370	SSP585	SSP126	SSP370	SSP585	SSP126	SSP370	SSP585
Lose	402.15	445.28	310.65	480.14	325.42	505.73	327.59	523.18	419.37
Gain	572.33	558.46	655.39	485.68	776.12	591.46	722.44	571.82	687.93
Unchanged	5217.85	4968.57	5105.61	4940.67	5091.84	4905.94	5093.76	4889.66	4995.85

## 4 Discussion

### 4.1 Dominant factors influencing the distribution of *Xanthium spinosum*

In this study, we used the MaxEnt model optimized through the R package ENMeval to simulate the potential distribution of *X. spinosum* under current and future periods (2040–2060, 2060–2080, and 2080–2100) across three climate scenarios (SSP126, SSP370, and SSP585). By comprehensively analyzing the contribution rates and training gain values of dominant climatic factors and plotting their response curves, we quantified the environmental characteristics of the natural distribution of *X. spinosum* and analyzed its suitable growth conditions. Results indicated that under current climatic conditions, temperature and precipitation are the predominant environmental variables that determine the distribution of *X. spinosum*, with temperature being particularly important as the dominant factor controlling its invasive spread (Gallardo et al., 2015). Among the environmental factors included in the model, Bio6 (Min Temperature of Coldest Month), Bio7 (Temperature Annual Range), Bio10 (Mean Temperature of Warmest Quarter), and Bio19 (Precipitation of Coldest Quarter) considerably influenced species distribution. Three of these four factors are temperature-related (Bio6, Bio7, Bio10), and Bio6 exhibited the highest contribution rate, indicating that temperature factors contribute more substantially to the geographical distribution of *X. spinosum* than precipitation. This is consistent with the ecological traits of the species including strong adaptability to desert environments and low water requirements (Andreani et al., 2017). Simulation predictions reveal that under future climate scenarios, Bio6 remains the dominant climatic factor that limits the potential suitable habitat for *X. spinosum*. Optimal seed germination for *X. spinosum* occurs near 25 °C (Auld, 1993), whereas germination of the congeneric *Xanthium strumarium* is inhibited above 35 °C and shows a sharp decline in germination rate (Saeed et al., 2020). Our response curve analysis is consistent with these findings: The optimal Mean Temperature of Warmest Quarter (Bio10) for *X. spinosum* growth is approximately 24.8 °C, and its survival threshold for Temperature Annual Range (Bio7) spans −22.4 °C to 33.2 °C, beyond which survival probability decreases. *X. spinosum* exhibits strong adaptability to Mediterranean climates (Andreani et al., 2017). Our results indicate that, under climate change, this species is likely to further expand into Mediterranean regions. As precipitation is concentrated during the cold season in most Mediterranean climates (Moore et al., 2020), and precipitation significantly influences seedling emergence, survival, and establishment of annual herbs (Wang and Gou, 2019), this study confirms that precipitation in the coldest quarter (Bio19) is a key environmental factor determining its distribution. In addition, *X. spinosum* exhibits broad tolerance for Precipitation of Coldest Quarter (Bio19) (69–458 mm), reflecting its strong adaptability to arid and humid environments, which is consistent with its inherent stress-tolerant traits (Tao et al., 2022).

### 4.2 Distribution pattern under current climatic conditions

This study reveals the dynamic response of suitable habitats for *X. spinosum* to climate change on a global scale. Currently, the core suitable habitat is concentrated in three regions: the western North America–central–western Europe corridor (120°W–60°W and 0°–60°E), the southeastern South America–West Africa transition zone (60°W–0° and 0°–20°E), and the southeastern Australia–East Asia coastal chain (120°E–180°E extending to 60°E–120°E), totaling 2,950.42×10^4^ km² (52.8% of the potential distribution area). This finding aligns closely with documented occurrence records (Güez et al., 2012; Lin et al., 2014), while simultaneously validating the reliability of MaxEnt for modeling the habitat suitability of *X. spinosum*. This broad adaptability enables establishment of the population in barren soils (e.g., moderately suitable areas in southeastern South America); however, the complete absence in core tropical rainforest zones (0°–10° latitude; unsuitable habitat covers 58.0% of these areas) indicates that high humidity and shaded environments constitute dispersal barriers. Furthermore, plant invasions are often closely associated with human activities. The seeds of *X. spinosum* possess a barbed structure on their surface; this facilitates unintentional dispersal through human activities and adheres to animal fur, enabling rapid expansion and colonization across extensive areas (Auld et al., 1988; Hocking and Liddle, 1986). In contrast, regions such as arid deserts, cold polar zones, and pristine tropical rainforests exhibit limited human activities owing to their extreme climates, complex topography, and remote location (Goosse et al., 2018; Zhang et al., 2018; Batterbury and Forsyth, 1999). Our findings also indicate that these regions for example the Sahara Desert and large parts of South American tropical rainforests, are classified as unsuitable distribution areas; this may also be related to the relatively low intensity of human activities in these regions.

### 4.3 Distribution patterns under future climate scenarios

Climate change impacts ecosystem stability by altering temperature and precipitation patterns, which potentially create new invasion opportunities for alien species, such as *X. spinosum*. Simulations using the optimized MaxEnt model indicate an expansion in the total suitable habitat area of *X. spinosum* across all future climate scenarios, which are consistent with predictions by Liu et al (Liu et al., 2025). for its distribution in China. Although total suitable area expands un0der all three scenarios, the core suitable habitat area decreases across all scenarios, particularly during early stages (e.g., reductions of 508.75×10^4^ km² under SSP126 and 548.76×10^4^ km² under SSP370). This phenomenon may relate to intensified climate change under global warming, including increased extreme heat events (Tang et al., 2025) and altered precipitation patterns (Rupp et al., 2022), which shifts environmental conditions away from the optimal growth requirements of *X. spinosum*, thereby contracting core habitats. These results are consistent with concerns that climate change exacerbates biodiversity loss and ecosystem vulnerability (Anderson, 2013; Marzloff et al., 2018).

*X. spinosum* is known for its high adaptability and reproductive capacity (Li and Ma, 2019). Its invasion into new regions poses severe management challenges (Gligor et al., 2022). The results of this study indicate that future contraction of suitable areas will be primarily concentrated in regions with high aridity and projected decreases in precipitation; for example, northern Kazakhstan and large parts of Africa (Teleubay et al., 2023; Nooni et al., 2022). As global warming exacerbates drought conditions, these areas may exceed the physiological tolerance range of *X. spinosum*, leading to a significant reduction in suitable habitats. In contrast, the expansion of suitable areas will occur mainly in high-latitude northern regions, such as western Russia, and areas where future precipitation is expected to increase, such as northwestern Australia (Watterson et al., 2016; Cullen and Grierson, 2007). Global climate warming, particularly the rise in the minimum temperature of the coldest month (bio6), is projected to facilitate the spread of *X. spinosum* into higher latitude regions. Consequently, particular attention should be given to the invasion risk of *X. spinosum* in northern high-latitude zones and other areas where future climate projections indicate that there will be both suitable temperatures and increased precipitation.

### 4.4 Limitations and future prospects

This study examines the suitable habitat patterns of *X. spinosum* by analyzing environmental factors and geographic distribution. Our model mainly incorporates climatic and soil variables, as climate is widely recognized as a key driver of species distribution (Pacifici et al., 2017), and soils in high-latitude regions may further constrain plant migration (Ni and Vellend, 2024). These variables are generally considered fundamental in shaping species distributions (Ulrich et al., 2014). However, there remains limited research on the biological interactions of *X. spinosum* and the effects of human activities under future climate scenarios. Despite this gap, the model developed in this study, based on environmental similarity principles (Elith et al., 2011), offers meaningful projections. Future studies should integrate biotic interactions and anthropogenic factors as predictors to better simulate potential invasion areas and provide more precise support for global biosecurity governance.

## 5 Conclusion

In this study, we used an optimized MaxEnt model to predict the global potential distribution pattern of *X. spinosum* under climate change. The main conclusions are as follows: The Min Temperature of Coldest Month (Bio6) is the core climatic factor determining the current global distribution of *X. spinosum* (contribution rate of 67.1%), with a suitable range of −7.3°C to 8.7°C. Under three future emission scenarios (SSP126, SSP370, and SSP585), Bio6 remains the most critical limiting factor. Under current climate conditions, the potential suitable habitat of *X. spinosum* is primarily distributed in the central to western regions of North America (120°W–60°W), the northern to southeastern regions of South America (60°W–0°), the central to western regions of Europe (0°–60°E), the central to western regions of Africa (0°–40°E), inland to coastal Asia (60°E–120°E), and southeastern Australia (120°E–180°E). The total area of the potential suitable habitat is approximately 5,620.18 × 10^4^ km², with the core suitable habitat covering 2,950.42 × 10^4^ km². Under three future climate scenarios, the potential suitable habitat area of *X. spinosum* shows an expanding trend, whereas the core suitable habitat area shows a shrinking trend. The significant reduction in future suitable areas may be related to the decreased precipitation in these regions.

## Data Availability

The original contributions presented in the study are included in the article/supplementary material. Further inquiries can be directed to the corresponding author.
